# Synthesis of Selenium-Quinone Hybrid Compounds with Potential Antitumor Activity via Rh-Catalyzed C-H Bond Activation and Click Reactions

**DOI:** 10.3390/molecules23010083

**Published:** 2017-12-30

**Authors:** Guilherme A. M. Jardim, Eduardo H. G. da Cruz, Wagner O. Valença, Daisy J. B. Lima, Bruno C. Cavalcanti, Claudia Pessoa, Jamal Rafique, Antonio L. Braga, Claus Jacob, Eufrânio N. da Silva Júnior

**Affiliations:** 1 Department of Chemistry, Institute of Exact Sciences, Federal University of Minas Gerais, UFMG, 31270-901 Belo Horizonte, Brazil; victimoffate18@gmail.com (G.A.M.J.); eduardoh_cruz@hotmail.com (E.H.G.d.C.); wagner.valenca1987@gmail.com (W.O.V.); 2 Division of Bioorganic Chemistry, Department of Pharmacy, University of Saarland, Campus B2 1, D-66123 Saarbruecken, Germany; 3 Department of Physiology and Pharmacology, Federal University of Ceará, CEP 60180-900 Fortaleza, Brazil; daisylima@gmail.com (D.J.B.L.); nunim_br@yahoo.com.br (B.C.C.); cpessoa@ufc.br (C.P.); 4 Department of Chemistry, Federal University of Santa Catarina, 88040-900 Florianópolis, Brazil; jamal.chm@gmail.com (J.R.); braga.antonio@ufsc.br (A.L.B.)

**Keywords:** lapachol, naphthoquinone, cancer, selenium, click chemistry, C-H activation

## Abstract

In continuation of our quest for new redox-modulating catalytic antitumor molecules, selenium-containing quinone-based 1,2,3-triazoles were synthesized using rhodium-catalyzed C-H bond activation and click reactions. All compounds were evaluated against five types of cancer cell lines: HL-60 (human promyelocytic leukemia cells), HCT-116 (human colon carcinoma cells), SF295 (human glioblastoma cells), NCIH-460 (human lung cells) and PC3 (human prostate cancer cells). Some compounds showed good activity with IC_50_ values below 1 µM. The cytotoxic potential of the naphthoquinoidal derivatives was also evaluated in non-tumor cells, exemplified by L929 cells. Overall, these compounds represent promising new lead derivatives and stand for a new class of chalcogenium-containing derivatives with potential antitumor activity.

## 1. Introduction

Cancer is considered one of the major leading causes of death worldwide, accounting for 8.8 million deaths in 2015, and responsible for one in six deaths globally [1]. It is a generic term for a large group of diseases characterized by abnormal cell growth which can invade adjoining parts of the body and general organs [2]. Each year, more than 14 million people are diagnosed with cancer, the majority of whom live in low- and middle-income countries [3]. Chemotherapy is one of the conventional treatments for patients diagnosed with cancer, together with radiation and surgery [4]. This systemic treatment consists in the circulation of anticancer drugs through the body by the blood circulatory system to reach cancer cells wherever they are, with the goal of annihilate both cancerous colonies and metastasized cancer cells within a patient’s body [5]. The side effects of chemotherapy are anemia, diarrhea, nausea, vomiting, hair loss and weakening of the immune system, as the actual anticancer drugs usually present low discrimination between normal and cancer cells [6].

Naphthoquinones are known to be widely distributed in Nature, occurring in animals [7], plants [8], and microorganisms [9]. This group of molecules is considered privileged structures in Medicinal Chemistry due to the variety of biological activities they display, such as antiallergic [10], antifungal [11], antiviral [12], antibacterial [13], and anti-inflammatory properties [14], among others [15,16,17]. Most of these activities are associated with the—often catalytic—generation of Reactive Oxygen Species (ROS) [18]. Among the various quinones, 1,4-naphthoquinones are well represented in cancer therapy by solid tumour anticancer drugs such as doxorubicin, mitomycin and mitoxantrone [19]. Considering natural quinones, we also find important cytotoxic 1,4-naphthoquinones, including lapachol [20], plumbagin [21] and shikonin [22].

Over the years our research group has concentrated its efforts on the investigation of the naturally occurring quinones from the tree species of the *Bignoniaceae* family, especially the ones extracted from the Ipe tree, like lapachol and *β*-lapachone [23]. The last one has a history of anticancer activity against various cancer molecular targets [24,25], being in Phase II clinical trials in the US for the treatment of solid tumors [26]. Derivatization of *β*-lapachone by insertion of the 1,2,3-triazole moiety proved to be an interesting strategy for enhancement of cytotoxic activity, and *β*-lapachone-based 1,2,3-triazoles were obtained in moderate to high yields, showing significant activities against HCT-8 cell-lines [27].

The underlying mode of cytotoxic biological action of such quinones is rather interesting and in many aspects sets these redox active agents apart from conventional anticancer agents. Various studies have demonstrated that cancer cells exhibit an abnormal redox status associated with increased basal levels of ROS and alterations of the antioxidant systems [28]. The use of redox-modulating and often catalytic compounds therefore appears as an efficient therapeutic approach by taking advantage of this biochemical singularity to selectively target cancer cells, as long as normal cells maintain their own redox homeostasis by controlling the balance between ROS generation and elimination [29]. Indeed, normal cells often can tolerate a certain level of exogenous ROS whereas cancer cells cannot [30]. Eventually, this pre-existing difference between cancer cells on the one side, and normal cells on the other, may provide a certain degree of selectivity which would be most welcome in any targeted anticancer therapy.

**Scheme 1:**
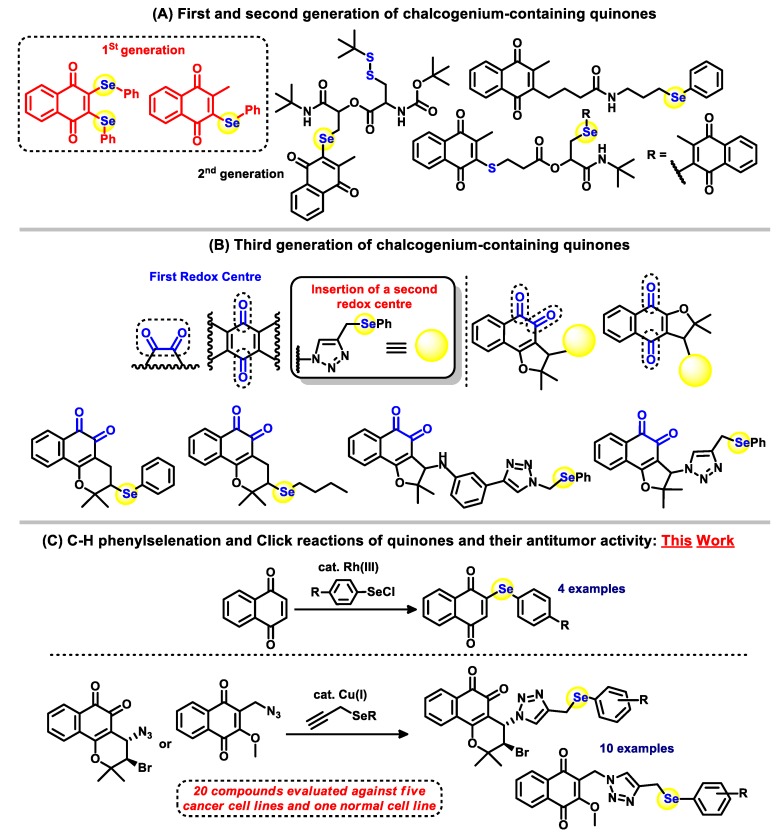
Overview of the different generations of hybrid redox molecules for the first and second generation (Panel (**A**)) to the third generation with lapachones (Panel (**B**)) and the products of Rh-activated C-H bond activation and click reactions (Panel (**C**)).

Following this notion of redox regulation in cancer cells by redox active small molecules, the strategy of combining—catalytic—*ROS generators* such as 1,4-naphthoquinones with *ROS modulators* such as selenium atoms in the same molecule for enhanced activity and selectivity has already been successfully explored by Jacob and co-workers a couple of years ago (Scheme 1A) [31,32,33]. These initial studies have confirmed that the selectivity of quinone compounds can be improved by these kinds of “molecular hybridization” without compromising the cytotoxicity of the compounds. Chalcogens, such as selenium and tellurium, are known to play a major role in the chemoprevention and possibly even in the treatment of cancer, a notion highlighted once more by the recent publications of Kieliszek, Lipinski, Błażejak and colleagues [34,35,36]. The choice of one or more chalcogen atoms as additional redox site(s) is therefore evident. Indeed, in fine agreement with these pioneering studies, our research group has recently described chalcogen-containing *β*-lapachones with remarkable activity against some cancer cell lines, including HL-60 with IC_50_ values of 0.94 μM and 0.53 μM and MOLT-4 (leukaemia) with IC_50_ values between 0.73 and 1.89 μM [37]. In previous work, selenium-containing *β*-lapachone-based 1,2,3-triazoles derivatives were also synthesized and exhibited high activity with IC_50_ values below 2 μM against cancer cells such as HL-60 and PC3 (human prostate) cells [38] (Scheme 1B).

Encouraged by these results, we now report the synthesis and biological evaluation of new selenium containing 1,4-naphthoquinone derivatives, obtained by two main synthetic strategies: C-H bond activation [39,40,41], and copper(I)-catalyzed alkyne-azide cycloaddition (CuAAC) [42,43,44,45] (Scheme 1C). Chalcogen-containing *β*-lapachones derivatives were also synthesized and their biological activity has been evaluated against cancer and normal cell lines.

## 2. Results and Discussion

### 2.1. Chemistry

**Scheme 2:**
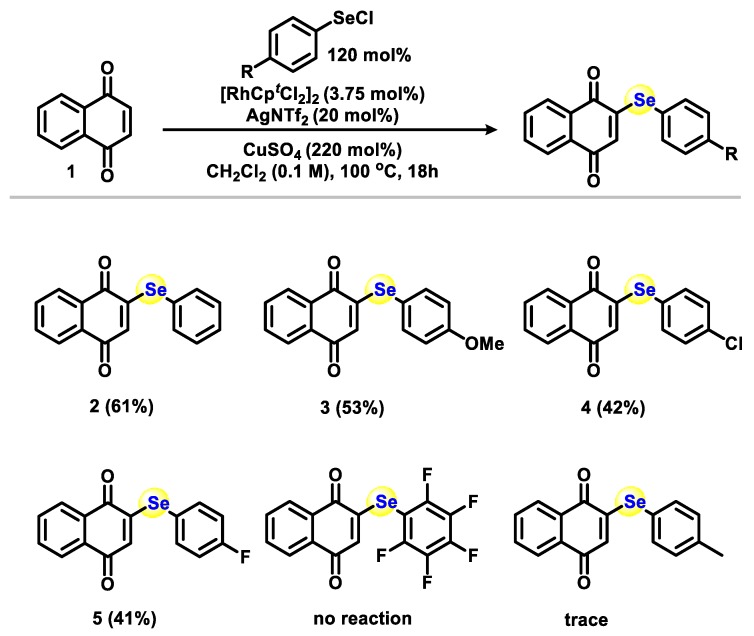
Functionalization of **1** via rhodium-catalyzed C-H activation.

Recently, our research group developed methodologies which enable the functionalization of quinones via rhodium catalyzed C-H activation [46,47], paving the way to new molecules containing iodine, bromine and selenium atoms with remarkable biological activity [48]. Encouraged by that fact, the same catalyst system was explored with other electrophilic selenium sources, focusing on obtaining new molecules with a substituent in the phenyl selenium moiety. 1,4-naphthoquinone (**1**) was reacted with substituted phenyl hypochloroselenoites and derivatives **2**–**5** were obtained with moderate yields. The reaction failed when electron deficient perfluorophenyl hypochloroselenoite was employed and only traces of product were observed for *p*-tolyl hypochloroselenoite (Scheme 2).

**Scheme 3:**
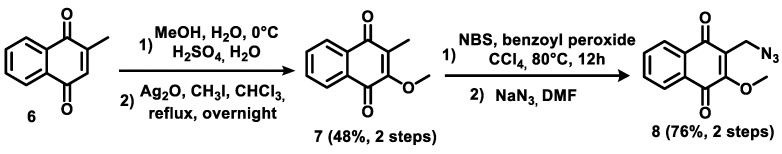
Synthetic pathway for obtaining azide derivative **8**.

Menadione (**6**) was reacted with hydrogen peroxide in methanol resulting in menadione oxide, that was promptly acidified with H_2_SO_4_ to obtain 2-hydroxy-3-methyl-1,4-naphthoquinone [49]. Methylation of the hydroxyl group was achieved by reaction with iodomethane and Ag_2_O in CHCl_3_ under reflux overnight, affording **7** in 48% over two steps. Next, **7** was reacted with NBS and benzoyl peroxide in CCl_4_ and bromination occurred in 76% yield [50]. Reaction with NaN_3_ in DMF gave **8** in quantitative yield (Scheme 3).

**Scheme 4:**
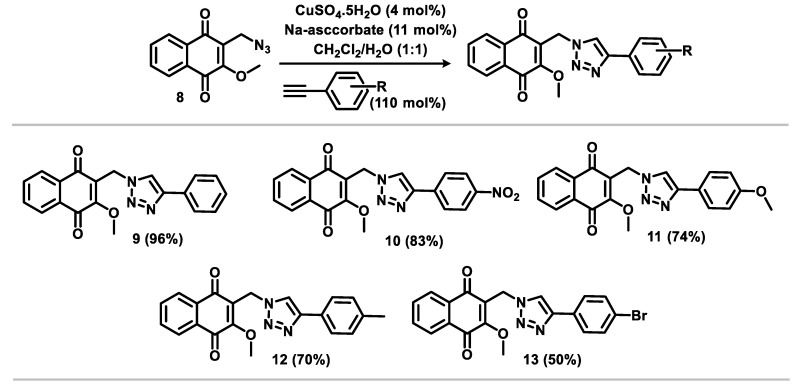
1,2,3-triazole derivatives obtained via classical copper(I) catalyzed “click” reaction.

Since 2002, copper-catalyzed “click” cycloadditions offer access to an almost unlimited array of triazole-containing structures [51]. For this reaction, copper(II) sulfate in aqueous media in the presence of sodium ascorbate, both in catalytic amounts, constitute the preferred catalytic system for this sort of transformation due to practicality, cost and robustness [52,53,54]. Besides more complicated multicomponent reactions, such as the Passerini or Ugi reaction, click reactions are ideally suited to assemble hybrid molecules, such as catalysts featuring two or more redox centers. Hence the second class of compounds was designed by adding a triazole portion in the 1,4-naphthoquinone backbone. In order to obtain 1,2,3-triazole derivatives, a classical copper(I) catalyzed “click” reaction [51,55] was employed to generate derivatives with substitution pattern in the 4-position of the benzene ring. Reaction with phenyl acetylene afforded **9** in 96% yield. Treatment of a more electron-deficient alkyne gave derivative **10** in excellent yield. Alkynes with electron donating groups, such as methoxy and methyl afforded derivatives **11** and **12** in good yields. Reaction with alkyne with presence of bromine in the benzene ring afforded **13** in moderate yield (Scheme 4).

**Scheme 5:**
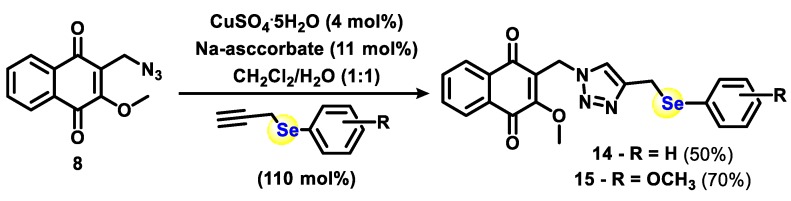
Selenium containing-1,2,3-triazole derivatives obtained via classical copper(I) catalyzed “click” reaction.

Next, in order to investigate the effect of a selenium atom in the bioactivity of the previous molecules, azide **8** was reacted with two different phenyl(prop-2-yn-1-yl)selanes substituted at the position 4 of the benzene ring (Scheme 5).

In contrast to menadione and its *para*-quinone derivatives, lapachones represent a class of highly reactive *ortho*-quinones and are renowned for their high activity against trypomastigote forms of *Trypanossoma cruzi* [56], the causative agent of Chagas disease. In this context, *β*-lapachone, in particular, is considered an effective substance against *Trypanossoma cruzi* [57]. This pathogenic microorganism seems to be rather sensitive to redox changes and therefore also to agents able to modulate its intracellular redox homeostasis. Recently, our research group has reported a novel selenium-containing *β*-lapachone derivative that showed remarkable activity against a series of cancer cell lines, proving that insertion of a second redox center contributes to an enhanced biological activity [38].

**Scheme 6:**
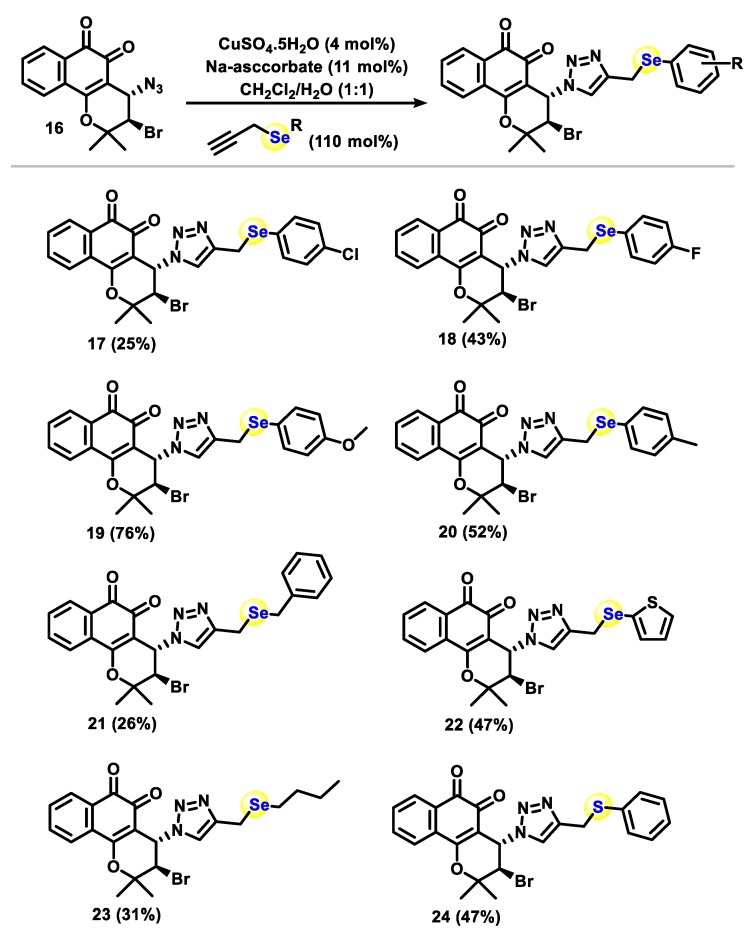
*β*-Lapachone selenium containing-1,2,3-triazoles. Note: Compounds were obtained in racemic form, but the stereochemistry *trans* was defined by X-ray crystallography as part of previous studies [27].

Motivated by those findings, *β*-lapachone-1,2,3-triazole-selenium derivatives were synthesized using CuAAc and were obtained in poor to good yields. Substituents at the aromatic ring of the selenium alkynes have shown to affect the yield of the reaction, and derivatives **17** and **18** containing chlorine and fluorine atoms in the position 4 of the aromatic ring were obtained in 25% and 43% yield, respectively. Electron-rich selenium alkyne bearing a methoxy group in the same position afforded derivative **19** in 76% yield. Interestingly, insertion of a methylene group in the alkyl chain and a less electron donating group such as methyl had a drastic effect on the reaction and derivatives **20** and **21** were obtained in moderate to poor yields. Modification of the benzene terminal group by a heterocyclic ring such as thiophene and an alkyl chain also influenced reactivity and derivatives **22** and **23** were obtained in 47% and 31% yield, respectively. Finally, exchanging the selenium atom by a sulfur one afforded derivative **24** in 47% yield (Scheme 6).

### 2.2. Biology

The various compounds synthesized as part of this study were subsequently evaluated for possible activity against different cancer cell lines, including HL-60, HCT-116, SF295, NCIH-460 and PC3 and one normal cell line, L929 (murine fibroblasts normal cells) (Table 1). Overall the IC_50_ and CI 95% values obtained reflect a considerable cytotoxicity associated with the quinones under investigation. Nonetheless, there are also some striking differences. Glancing over the data available so far, it is immediately apparent that the lapachone derivatives exhibit a considerably higher activity when compared to the menadiones. Whilst the IC_50_ values for the 1,4-quinones are usually between 10 and 100 μM, the lapachone derivatives are active well below this, generally between 1 and 10 μM, with some of the most active lapachones, such as compound **18**, even active in sub-micromolar concentrations. Indeed, this compound exhibits IC_50_ values of 0.59 μM, 0.37 μM and 0.36 μM against HL-60, HCT-116 and L929 cell lines, which are in the range of the ones measured for the benchmark anticancer drug doxorubicin (0.21 μM and 1.72 μM against HCT-116 and L929 cell lines, respectively).

Interestingly, the exceptional activity of compound **18** seems to result from a combination of (a) the lapachone; (b) selenium and (c) an electron-withdrawing substituent in *para*-position to Se which may render this chalcogen more (re-)active. Although the data available to date is very limited, and hence any structure-reactivity relationship is still highly speculative, there is some support for this notion. First of all, the menadione-derivatives, as mentioned already, are considerably less active, often with a factor of ten or even more between their activities and the ones of the lapachones. Secondly, compound **24**, which contains a sulfur instead of selenium, is the least active of the lapachone derivatives tested—although its activity still compares favourably with the ones of the menadione derivatives. And finally, the electron-donating analogues of compound **18** are also less active, although the differences are minor.

Apart from the considerable activity of some of the compounds presented, which compares well with the one of the common drug doxorubicin, there is also some selectivity against certain cancer cell lines. These differences in IC_50_ values are minor yet notable. Still, there is no significant “selective cytotoxicity” against one particular cell line, which is somewhat disappointing yet not entirely unexpected, as none of the cell lines employed in this study represents truly “normal human cells”.

## 3. Materials and Methods

### 3.1. General Information

Starting materials available from commercial suppliers were used as received, unless stated otherwise. All reagents requiring purification were purified using standard laboratory techniques, according to methods published by Perrin, Armarego, and Perrin (Pergamon Press, 1966). Catalytic reactions were performed under an atmosphere of dry nitrogen or argon; glassware, syringes and needles were either flame dried immediately prior to use or placed in an oven (200 °C), for at least 2 h, and allowed to cool either in a desiccator or under an atmosphere of nitrogen or argon; liquid reagents, solutions or solvents were added via syringe through rubber septa; solid reagents were added inside a glovebox. Column chromatography was performed on silica gel (SiliaFlash G60 UltraPure 60–200 μm, 60 Å, Quebec City, QC, Canada). ^1^H- and ^13^C-NMR were recorded at room temperature using an AVANCE DRX200 or an DRX400 MHz instrument (Bruker, Billerica, MA, USA) in the solvents indicated. Chemical shifts (*δ*) are given in parts per million (ppm) and coupling constants (*J*) in Hertz (Hz). All assignments of NMR spectra were based on 2D NMR data (DEPT-135, COSY, HSQC and HMBC). Mass spectra were recorded using a FT-ICRMS Apex 4e 7.0T FT-MS (ESI^+^ mode) (Bruker Daltonics) or a GCMS QP2010+ (EI^+^ mode) instrument (Shimadzu). Infrared spectra were recorded on a Spectrum One FTIR spectrometer (Perkin Elmer, 710 Bridgeport Avenue, Shelton, CT, USA) as thin films or solids compressed on a diamond plate and are uncorrected. Data were processed employing Bruker Data Analysis software version 4.0. Compounds were named following IUPAC rules as applied by ChemBioDraw Ultra (version 12.0).

1,4-Naphthoquinone (**1**) and menadione (**6**) were purchased from Sigma Aldrich (St. Louis, MI, USA), purified via reduced pressure sublimation using a cold finger sublimation apparatus (50 °C, 0.9 mbar) and stored in a glovebox to prevent contact with moisture. Lapachol was extracted from the heartwood of *Tabeluia* sp. (Tecoma). A saturated aqueous sodium carbonate solution was added to the sawdust of ipe tree. Upon observing rapid formation of lapachol sodium salt, hydrochloric acid was added, allowing the precipitation of lapachol. Subsequently, the solution was filtered and a yellow solid was obtained. This solid was purified by recrystallizations with hexane. *β*-Lapachone was synthesised from lapachol by cyclization with concentrated sulfuric acid.

#### 3.1.1. General Procedure for Phenylselenation at the 2-Position

An oven dried re-sealable tube was charged with 1,4-naphthoquinone (**1**) (15.8 mg, 0.10 mmol), [RhCp*^t^*Cl_2_]_2_ (3.75 mol %, 2.6 mg), silver *bis*(trifluoromethanesulfonyl)imide (20 mol %, 7.8 mg), anhydrous copper sulfate (220 mol %, 0.22 mmol, 34.9 mg) and the corresponding substituted phenylselenyl chloride (120 mol %, 0.12 mmol). The tube was connected to a Schlenk line and an inert atmosphere was achieved. Anhydrous CH_2_Cl_2_ (1 mL) was added via a syringe and the tube was sealed. The mixture was heated at 100 °C for 18 h. After cooling, the mixture was filtered through a pad of Celite and purified via column chromatography (toluene) under the conditions noted.

*2-(Phenylselanyl)naphthalene-1,4-dione* (**2**): Phenylselenyl chloride (23.0 mg, 0.12 mmol) was used as substrate. The product was obtained in 61% yield as red crystals; m.p. (°C) = 152.8–153.7; IR (solid, cm^−1^) *ν*: 2930 (w), 1664 (s), 1296 (m), 690 (s); HRMS (EI^+^): 313.9848 [M]^+^. Cald. for [C_16_H_10_SeO_2_]: 313.9846; ^1^H-NMR (400 MHz, CDCl_3_) *δ*: 8.14 (dd, *J* = 7.2, 1.9 Hz, 1H), 8.03 (dd, *J* = 6.1, 1.9 Hz, 1H), 7.82–7.67 (m, 2H), 7.68–7.61 (m, 2H), 7.57–7.42 (m, 3H), 6.40 (s, 1H); ^13^C-NMR (100 MHz, CDCl_3_) *δ*: 183.0, 181.7, 157.0, 137.1, 134.3, 133.3, 132.8, 132.3, 131.6, 130.4, 130.2, 126.9, 126.7, 124.4.

*2-((4-Methoxyphenyl)selanyl)naphthalene-1,4-dione* (**3**): 4-Methoxyphenylselenyl chloride (26.6 mg, 0.12 mmol) was used. The product was obtained in 53% yield as an orange solid; m.p. (°C) = 140.7–148.3; IR (solid, cm^−1^) *ν*: 2921 (w), 1641 (s), 1246 (m), 694 (w); HRMS (ESI^+^): 345.0015 [M + H]^+^. Cald. for [C_17_H_13_SeO_3_]^+^: 345.0024; ^1^H-NMR (400 MHz, CDCl_3_) *δ*: 8.14 (d, *J* = 6.9 Hz, 1H), 8.05 (d, *J* = 7.1 Hz, 1H), 7.77–7.69 (m, 2H), 7.54 (d, *J* = 8.6 Hz, 2H), 7.00 (d, *J* = 8.6 Hz, 2H), 6.40 (s, 1H), 3.89 (s, 3H). ^13^C-NMR (100 MHz, CDCl_3_) *δ*: 183.1, 181.7, 161.3, 157.7, 138.6, 134.3, 133.3, 132.8, 132.4, 131.8, 126.9, 126.7, 116.2, 114.5, 55.4.

*2-((4-Chlorophenyl)selanyl)naphthalene-1,4-dione* (**4**): 4-Chlorophenylselenyl chloride (27.1 mg, 0.12 mmol) was used. The product was obtained in 42% yield as an orange solid; m.p. (°C) = 154.8–155.7; IR (solid, cm^−1^) *ν*: 2917 (m), 1648 (s), 1085 (m), 694 (m); HRMS (ESI^+^): 348.9515 [M + H]^+^. Cald. for [C_16_H_10_ClSeO_2_]^+^: 348.9529; ^1^H-NMR (400 MHz, CDCl_3_) *δ*: 8.12 (dd, *J* = 6.7, 2.1 Hz, 1H), 8.04 (dd, *J* = 6.7, 2.1 Hz, 1H), 7.78–7.68 (m, 2H), 7.57 (d, *J* = 8.5 Hz, 2H), 7.44 (d, *J* = 8.5 Hz, 2H), 6.37 (s, 1H). ^13^C-NMR (100 MHz, CDCl_3_) *δ*: 182.9, 181.6, 156.4, 138.4, 137.0, 134.4, 133.4, 132.8, 132.30, 131.6, 130.7, 127.0, 126.8, 122.6.

*2-((4-Fluorophenyl)selanyl)naphthalene-1,4-dione* (**5**): 4-Fluorophenylselenyl chloride (25.1 mg, 0.12 mmol) was used. The product was obtained in 41% yield as an orange solid; m.p. (°C) = 139.2–139.9; IR (solid, cm^−1^) *ν*: 2924 (w), 1651 (s), 1243 (m), 690 (w); HRMS (EI^+^): 332.9810 [M + H]^+^. Cald. for [C_16_H_10_FSeO_2_]^+^: 332.9825; ^1^H-NMR (400 MHz, CDCl_3_) *δ*: 8.13 (dd, *J* = 7.3, 1.7 Hz, 1H), 8.04 (dd, *J* = 7.3, 1.7 Hz, 1H), 7.78–7.68 (m, 2H), 7.61 (dd, *J* = 8.6, 5.4 Hz, 2H), 7.17 (t, *J* = 8.6 Hz, 2H), 6.35 (s, 1H). ^13^C-NMR (100 MHz, CDCl_3_) *δ*: 182.9, 181.6, 165.3, 162.8, 156.8, 139.26 (d, *J* = 8.3 Hz), 134.4, 133.4, 132.8, 132.3, 131.6, 126.86 (d, *J* = 13.7 Hz), 119.24 (d, *J* = 3.6 Hz), 117.84 (d, *J* = 21.8 Hz).

*2-Hydroxy-3-methylnaphthalene-1,4-dione* [49] (*menadione*, **6**): (1.0 g; 5.8 mmol) was dissolved in 10 mL methanol and chilled on an ice bath. A solution of 0.2 g of anhydrous Na_2_CO_3_ and 1 mL of 30% H_2_O_2_ in 5 mL of water were added slowly with the reaction mixture maintained at 0 °C. Then 100 mL of water were added and 2-methyl-1,4-naphthoquinone oxide precipitated as colourless crystals, which were collected by filtration and dried. The solid was treated with 5 mL concentrated H_2_SO_4_ and the mixture was allowed to stand for 10 min. Subsequent addition of 20 mL water resulted in a yellow precipitate which was filtered, dried in vacuum and purified by column chromatography (EtOAc/hexane 1:4). The product was obtained in 78% yield as yellow needles; m.p. (°C) = 178.2–179.1; IR (solid, cm^−1^) *ν*: 3332 (s), 1657 (s), 1276 (m), 726 (s); HRMS (EI^+^): 188.0467 [M]^+^. Cald. for [C_11_H_8_O_3_]: 188.0473; ^1^H-NMR (400 MHz, CDCl_3_) δ: 8.13 (dd, *J* = 7.6, 1.2 Hz, 1H), 8.08 (dd, *J* = 7.6, 1.2 Hz, 1H), 7.75 (td, *J* = 7.5, 1.3 Hz, 1H), 7.68 (td, *J* = 7.5, 1.3 Hz, 1H), 7.33 (s, 1H), 2.11 (s, 3H). ^13^C-NMR (100 MHz, CDCl_3_) δ: 185.0, 181.2, 153.1, 134.8, 132.9, 132.9, 129.4, 126.7, 126.1, 120.5, 8.7.

*2-Methoxy-3-methylnaphthalene-1,4-dione* (**7**): To a solution of 2-hydroxy-3-methylnaphthalene-1,4-dione (188.2 mg, 1 mmol) in CHCl_3_ (30 mL) were added Ag_2_O (463.5 mg, 2.0 mmol) and iodomethane (125.0 μL, 2 mmol). The reaction was refluxed for 48 h, filtered through a pad of Celite and the solvent was removed under reduced pressure. The residue was purified via column chromatography (EtOAc/hexane 1:5). The product was obtained in 62% yield as a yellow solid; m.p. (°C) = 99.3–100.7; IR (solid, cm^−1^) *ν*: 2953 (w), 1674 (s), 1269 (m), 720 (s); HRMS (EI^+^): 202.0627 [M]^+^. Cald. for [C_12_H_10_O_3_]: 202.0630; ^1^H-NMR (400 MHz, CDCl_3_) δ: 8.20–7.89 (m, 2H), 7.84–7.58 (m, 2H), 4.12 (s, 3H), 2.09 (s, 3H). ^13^C-NMR (100 MHz, CDCl_3_) δ: 185.7, 181.2, 157.8, 133.7, 133.2, 132.0, 131.69, 131.5, 126.1, 126.1, 61.0, 9.2.

*2-(Bromomethyl)-3-methoxynaphthalene-1,4-dione*: 2-(Bromomethyl)-3-methoxynaphthalene-1,4-dione was prepared as described in reference [50]. To a stirred solution of 2-methoxy-3-methyl-1,4-naphthoquinone (**7**) (1.0 g, 5 mmol) in CCl_4_ (30 mL) were added NBS (900 mg, 5.0 mmol) and benzoyl peroxide (0.5 g, 2.1 mmol). The mixture was stirred under white light irradiation (60 W) at 80 °C for 12 h. The solvent was then removed *in vacuo* and the residue was dissolved in CH_2_Cl_2_ (60 mL) and washed with H_2_O (4 × 30 mL). The organic layer was dried with Na_2_SO_4_, filtered, evaporated and purified by column chromatography (EtOAc/hexane 1:4). The product was obtained in 76% yield as a yellow solid; m.p. (°C) = 117.4–118.7; IR (solid, cm^−1^) *ν*: 2953 (w), 1677 (s), 1217 (s), 723 (s); ^1^H-NMR (400 MHz, CDCl_3_) δ: 8.12 (dd, *J* = 6.8, 2.2 Hz, 1H), 8.06 (dd, *J* = 6.8, 2.2 Hz, 1H), 7.78–7.70 (m, 2H), 4.48 (s, 2H), 4.35 (s, 3H). ^13^C-NMR (100 MHz, CDCl_3_) δ: 182.7, 181.3, 158.1, 134.3, 133.6, 131.6, 131.5, 129.1, 126.5, 126.4, 61.7, 20.0.

*2-(Azidomethyl)-3-methoxynaphthalene-1,4-dione* (**8**): To a stirred solution of 2-(bromomethyl)-3-methoxynaphthalene-1,4-dione (281.1 mg, 1 mmol) in DMF (5 mL) was added NaN_3_ (195.0 mg, 3 mmol). The reaction was allowed to stir for 20 min and quenched with H_2_O (15 mL). Then, the mixture was extracted with ethyl acetate (30 mL), and the organic phase was separated and dried with Na_2_SO_4_. The solvent was removed under vacuum and the residue was purified via column chromatography (EtOAc/Hexane 1:5). The product was obtained in 100% yield as a dark yellow solid; m.p. (°C) = 69.1–70.0; IR (solid, cm^−1^) *ν*: 2957 (w), 2100 (s), 1677 (s), 1273 (s), 730 (s); MS (ESI^+^): 244.1 [M + H]^+^. Cald. for [C_12_H_10_ N_3_O_3_]^+^: 244.1; ^1^H-NMR (400 MHz, CDCl_3_) δ: 8.05 (dd, *J* = 6.4, 2.5 Hz, 1H), 8.01 (dd, *J* = 6.4, 2.5 Hz, 1H), 7.75–7.66 (m, 2H), 4.30 (s, 2H), 4.25 (s, 3H). ^13^C-NMR (100 MHz, CDCl_3_) δ: 184.0, 181.4, 158.9, 134.3, 133.6, 131.5, 131.4, 126.4, 126.0, 61.9, 42.7.

#### 3.1.2. General Procedure for the Synthesis of Triazoles

The corresponding alkynes (1.1 mmol) were reacted with 2-(azidomethyl)-3-methoxy-naphthalene-1,4-dione (**8**) (243.2 mg, 1 mmol) or (3*R*,4*S*)-4-azido-3-bromo-2,2-dimethyl-3,4-dihydro-2*H*-benzo[*h*]chromene-5,6-dione (**18**) (362.2 mg, 1 mmol) in 12 mL of CH_2_Cl_2_/H_2_O (1:1) using CuSO_4_.5H_2_O (9.3 mg, 0.04 mmol) and sodium ascorbate (22 mg, 0.11 mmol) as the catalytic system. The mixture was stirred at room temperature and monitored by TLC until total consumption of starting material. The mixture was extracted with CH_2_Cl_2_, dried with Na_2_SO_4_ and concentrated under reduced pressure. The resulting residue was purified by column chromatography on silica gel using a gradient mixture of hexane/ethyl acetate with increasing polarity up to 100% ethyl acetate as eluent.

*2-Methoxy-3-((4-phenyl-1H-1,2,3-triazol-1-yl)methyl)naphthalene-1,4-dione* (**9**): Ethynylbenzene (112.3 mg, 1.1 mmol) was used. The product was obtained in 96% yield as a dark yellow solid; m.p. (°C) = 103.8–104.7; IR (solid, cm^−1^) *ν*: 2921 (m), 1671 (s), 1263 (m), 763 (s); HRMS (ESI^+^): 346.1160 [M + H]^+^. Cald. for [C_20_H_16_N_3_O_3_]^+^: 346.1186; ^1^H-NMR (400 MHz, CDCl_3_) δ: 8.09–7.99 (m, 2H), 7.95 (s, 1H), 7.79 (d, *J* = 7.4 Hz, 2H), 7.75–7.64 (m, 2H), 7.37 (t, *J* = 7.4 Hz, 2H), 7.28 (t, *J* = 7.4 Hz, 1H), 5.55 (s, 2H), 4.30 (s, 3H). ^13^C-NMR (100 MHz, CDCl_3_) δ: 184.0, 181.3, 159.4, 147.5, 134.4, 133.8, 131.5, 131.3, 130.7, 128.7, 128.0, 126.5, 126.4, 125.7, 125.1, 120.8, 62.1, 42.3, 29.7.

*2-Methoxy-3-((4-(4-nitrophenyl)-1H-1,2,3-triazol-1-yl)methyl)naphthalene-1,4-dione* (**10**): 1-Ethynyl-4-nitrobenzene (161.8 mg, 1.1 mmol) was used. The product was obtained in 83% yield as a bright yellow solid; m.p. (°C) = 205.8–206.7; IR (solid, cm^−1^) *ν*: 3134 (w), 2924 (w), 1667 (s), 1338 (m), 756 (w); HRMS (ESI^+^): 391.1010 [M + H]^+^. Cald. for [C_20_H_15_N_4_O_5_]^+^: 391.1037; ^1^H-NMR (400 MHz, CDCl_3_) δ: 8.26 (d, *J* = 8.5 Hz, 2H), 8.14 (s, 1H), 8.09 (t, *J* = 6.5 Hz, 2H), 7.99 (d, *J* = 8.5 Hz, 2H), 7.81–7.70 (m, 2H), 5.62 (s, 2H), 4.37 (s, 3H). ^13^C--NMR (100 MHz, CDCl_3_) δ: 184.1, 181.3, 159.5, 147.3, 136.9, 134.6, 133.9, 131.5, 131.2, 126.7, 126.4, 126.1, 124.4, 124.2, 122.4, 62.3, 42.6, 29.7.

*2-Methoxy-3-((4-(4-methoxyphenyl)-1H-1,2,3-triazol-1-yl)methyl)naphthalene-1,4-dione* (**11**): 1-Ethynyl-4-methoxybenzene (145.4 mg, 1.1 mmol) was used. The product was obtained in 74% yield as an yellow solid; m.p. (°C) = 149.3–150.7; IR (solid, cm^−1^) *ν*: 3088 (w), 1671 (s), 1266 (m), 720 (w); HRMS (ESI^+^): 376.1370 [M + H]^+^. Cald. for [C_21_H_18_N_3_O_4_]^+^: 376.1292; ^1^H-NMR (400 MHz, CDCl_3_) δ: 8.12–7.98 (m, 2H), 7.91 (s, 1H), 7.76–7.66 (m, 4H), 7.19 (d, *J* = 7.9 Hz, 2H), 5.55 (s, 2H), 4.31 (s, 3H), 2.35 (s, 3H). ^13^C-NMR (100 MHz, CDCl_3_) δ: 184.1, 181.3, 159.4, 147.6, 137.8, 134.4, 133.7, 131.5, 131.3, 129.4, 127.9, 126.5, 126.4, 125.6, 125.2, 120.4, 62.1, 42.2, 21.2.

*2-Methoxy-3-((4-(p-tolyl)-1H-1,2,3-triazol-1-yl)methyl)naphthalene-1,4-dione* (**12**): 1-Ethynyl-4-methylbenzene (127.8 mg, 1.1 mmol) was used. The product was obtained in 70% yield as an yellow solid; m.p. (°C) = 123.1–124.5; IR (solid, cm^−1^) *ν*: 3112 (w), 1644 (s), 1217 (m), 819 (s); HRMS (ESI^+^): 360.134 [M + H]^+^. Cald. for [C_21_H_18_N_3_O_3_]^+^: 360.1343; ^1^H-NMR (400 MHz, CDCl_3_) δ: 8.09–8.00 (m, 2H), 7.86 (s, 1H), 7.78–7.65 (m, 4H), 6.90 (d, *J* = 8.7 Hz, 2H), 5.54 (s, 2H), 4.30 (s, 3H), 3.81 (s, 3H). ^13^C-NMR (100 MHz, CDCl_3_) δ: 184.1, 181.3, 159.5, 159.4, 147.4, 134.4, 133.7, 131.5, 131.3, 127.0, 126.5, 126.4, 125.2, 123.4, 120.0, 114.2, 62.1, 55.3, 42.2.

*2-((4-(4-Bromophenyl)-1H-1,2,3-triazol-1-yl)methyl)-3-methoxynaphthalene-1,4-dione* (**13**): 1-Bromo-4-ethynylbenzene (199.1 mg, 1.1 mmol) was used. The product was obtained in 50% yield as an orange solid; m.p. (°C) = 174.1–175.0; IR (solid, cm^−1^) *ν*: 2914 (w), 1674 (s), 1213 (m), 822 (s); HRMS (ESI^+^): 425.9820 [M+2]^+^. Cald. for [C_20_H_16_BrN_3_O_3_]^+^: 425.0364; ^1^H-NMR (400 MHz, CDCl_3_) δ: 8.15–8.01 (m, 2H), 7.96 (s, 1H), 7.79–7.71 (m, 2H), 7.69 (d, *J* = 8.4 Hz, 2H), 7.52 (d, *J* = 8.4 Hz, 2H), 5.58 (s, 2H), 4.34 (s, 3H). ^13^C-NMR (100 MHz, CDCl_3_) δ: 184.1, 181.3, 159.4, 146.6, 134.5, 133.8, 131.9, 131.5, 131.3, 129.6, 127.2, 126.6, 126.4, 124.9, 121.9, 120.9, 62.2, 42.4.

*2-Methoxy-3-((4-((phenylselanyl)methyl)-1H-1,2,3-triazol-1-yl)methyl)naphthalene-1,4-dione* (**14**): Phenyl(prop-2-yn-1-yl)selane (214.6 mg, 1.1 mmol) was used. The product was obtained in 50% yield as an bright yellow solid; m.p. (°C) = 69–71; IR (solid, cm^−1^) *ν*: 2921 (w), 1679, 1645 (s), 964, 734 (m); HRMS (ESI^+^): 440.0520 [M + H]^+^. Cald. for [C_21_H_18_N_3_O_3_Se]^+^: 440.0508; ^1^H-NMR (400 MHz, CDCl_3_) δ: 8.09–8.03 (m, 2H), 7.77–7.71 (m, 2H), 7.43 (dd, *J* = 7.8, 1.6 Hz, 2H), 7.37 (s, 1H), 7.18–7.10 (m, 3H), 5.43 (s, 2H), 4.24 (s, 3H), 4.14 (s, 2H). ^13^C-NMR (100 MHz, CDCl_3_) δ: 183.8, 181.3, 159.2, 145.2, 134.5, 133.8, 133.3, 131.5, 131.3, 129.9, 129.0, 127.3, 126.5, 126.4, 125.0, 122.8, 62.0, 42.1, 20.6.

*2-Methoxy-3-((4-(((4-methoxyphenyl)selanyl)methyl)-1H-1,2,3-triazol-1-yl)methyl)naphthalene-1,4-dione* (**15**): (4-Methoxyphenyl)(prop-2-yn-1-yl)selane (247.7 mg, 1.1 mmol) was used. The product was obtained in 70% yield as a dark yellow oil; IR (oil, cm^-1^) *ν*: 2957 (w), 1677 (s), 1243 (m), 726 (s); MS (ESI^+^): 440.0 [M + H]^+^. Cald. for [C_21_H_18_ N_3_O_3_Se]^+^: 440.0; ^1^H-NMR (400 MHz, CDCl_3_) δ: 8.08–8.03 (m, 2H), 7.77–7.70 (m, 2H), 7.37 (s, 1H), 7.34 (d, *J* = 8.6 Hz, 2H), 6.71 (d, *J* = 8.6 Hz, 2H), 5.43 (s, 2H), 4.26 (s, 3H), 4.04 (s, 2H), 3.73 (s, 3H). ^13^C-NMR (100 MHz, CDCl_3_) δ: 183.9, 181.4, 159.6, 159.3, 145.5, 136.3, 134.5, 133.8, 131.5, 131.3, 126.6, 126.4, 125.0, 122.8, 119.7, 114.8, 62.1, 55.27, 42.2, 21.6.

*3-Bromo-4-(4-(((4-chlorophenyl)selanyl)methyl)-1H-1,2,3-triazol-1-yl)-2,2-dimethyl-3,4-dihydro-2H-benzo[h]chromene-5,6-dione* (**17**): (4-Chlorophenyl)(prop-2-yn-1-yl)selane (252.5 mg, 1.1 mmol) was used. The product was obtained in 25% yield as a yellow powder. m.p. (°C) = 185.3–187.1, IR (solid, cm^−1^) *ν*: 1656 (s), 782 (w), HRMS (EI^+^): 591.9556 [M]^+^. Cald. for [C_16_H_10_SeO_2_]: 591.9536; ^1^H-NMR (400 MHz, CDCl_3_) *δ*: 8.10 (d, *J* = 7.6 Hz, 1H), 7.90 (d, *J* = 7.6 Hz, 1H), 7.74 (t, *J* = 7.2 Hz, 1H), 7.63 (t, *J* = 7.2 Hz, 1H), 7.54 (s, 1H), 7.41 (d, *J* = 8.4 Hz, 2H), 7.22 (d, *J* = 8.4 Hz, 2H), 5.56 (d, *J* = 9.2 Hz, 1H), 4.90 (d, *J* = 9.1 Hz, 1H), 4.12 (s, 2H), 1.75 (s, 3H), 1.64 (s, 3H). ^13^C-NMR (100 MHz, CDCl_3_) *δ*: 177.8, 176.4, 162.6, 135.7, 135.2, 134.0, 132.3, 130.6, 130.5, 129.2, 129.2, 127.3, 125.2, 125.0, 110.4, 83.4, 58.9, 54.5, 27.5, 21.0, 20.5.

*3-Bromo-4-(4-(((4-fluorophenyl)selanyl)methyl)-1H-1,2,3-triazol-1-yl)-2,2-dimethyl-3,4-dihydro-2H-benzo[h]chromene-5,6-dione* (**18**): (4-Fluorophenyl)(prop-2-yn-1-yl)selane (234.4 mg, 1.1 mmol) was used. The product was obtained in 43% yield as an orange powder. m.p. (°C) = 187.1–190.0, IR (solid, cm^−1^) *ν*: 1660 (s), 1214 (w), HRMS (EI^+^): 575.9885 [M]^+^. Cald. for [C_16_H_10_SeO_2_]: 575.9837; ^1^H-NMR (400 MHz, CDCl_3_) *δ*: 8.08 (d, *J* = 7.5 Hz, 1H), 7.89 (d, *J* = 7.7 Hz, 1H), 7.73 (t, *J* = 7.5 Hz, 1H), 7.62 (t, *J* = 7.5 Hz, 1H), 7.52 (s, 1H), 7.36 (s, 1H), 7.46 (dd, *J* = 8.4, 5.6 Hz, 2H), 6.95 (t, *J* = 8.7 Hz, 2H), 5.57 (d, *J* = 9.1 Hz, 1H), 4.90 (d, *J* = 9.1 Hz, 1H), 4.09 (s, 2H), 1.75 (s, 3H), 1.64 (s, 3H). ^13^C-NMR (100 MHz, CDCl_3_) *δ*: 178.0, 176.6, 164.2, 162.8, 161.7, 144.6, 137.2 (d, *J* = 8.0 Hz), 135.4, 132.5, 130.8, 130.7, 129.4, 125.3 (d, *J* = 32.7 Hz), 123,8 (d, *J* = 3.4 Hz) 116.5 (d, *J* = 21.4 Hz), 110.6, 83.7, 59.1, 54.8, 27.7, 21.6, 20.7.

*3-Bromo-4-(4-(((4-methoxyphenyl)selanyl)methyl)-1H-1,2,3-triazol-1-yl)-2,2-dimethyl-3,4-dihydro-2H-benzo[h]chromene-5,6-dione* (**19**): (4-Methoxyphenyl)(prop-2-yn-1-yl)selane (247.6 mg, 1.1 mmol) was used. The product was obtained in 76% yield as an orange powder. m.p. (°C) = 113.5–114.6, IR (solid, cm^−1^) *ν*: 1654 (s), 1246 (w), HRMS (EI^+^): 588.0093 [M]^+^. Cald. for [C_16_H_10_SeO_2_]: 588.0032, ^1^H-NMR (400 MHz, CDCl_3_) *δ*: 8.11 (d, *J* = 7.6 Hz, 1H), 7.91 (d, *J* = 7.8 Hz, 1H), 7.75 (t, *J* = 7.6 Hz, 1H), 7.64 (t, *J* = 7.6 Hz, 1H), 7.74 (d, *J* = 9.4 Hz, 3H), 6.81 (d, *J* = 8.5 Hz, 2H), 5.58 (d, *J* = 9.0 Hz, 1H), 4.93 (d, *J* = 9.0 Hz, 1H), 4.06 (s, 2H), 3.79 (s, 3H), 1.75 (s, 3H), 1.66 (s, 3H). ^13^C-NMR (100 MHz, CDCl_3_) *δ*: 178.1, 176.6, 162.8, 159.9, 144.9, 137.2, 135.4, 132.5, 130.8, 130.7, 129.2, 125.5, 124.9, 119.3, 115.0, 110.5, 83.6, 58.9, 55.4, 54.8, 21.8, 21.0, 14.4.

*4-(4-((Benzylselanyl)methyl)-1H-1,2,3-triazol-1-yl)-3-bromo-2,2-dimethyl-3,4-dihydro-2H-benzo[h]-chromene-5,6-dione* (**20**): Benzyl(prop-2-yn-1-yl)selane (230.1 mg, 1.1 mmol) was used. The product was obtained in 52% yield as a yellow powder. m.p. (°C) = 112.2–115.6, IR (solid, cm^−1^) *ν*: 1656 (s), HRMS (EI^+^): 572.0089 [M]^+^. Cald. for [C_16_H_10_SeO_2_]: 572.0083; ^1^H-NMR (400 MHz, CDCl_3_) *δ*: 8.10 (d, *J* = 7.6 Hz, 1H), 7.90 (d, *J* = 7.7 Hz, 1H), 7.74 (t, *J* = 7.6 Hz, 1H), 7.63 (t, *J* = 7.5 Hz, 1H), 7.47 (s, 1H), 7.39 (d, *J* = 8.0 Hz, 2H), 7.06 (d, *J* = 7.7 Hz, 2H), 5.56 (d, *J* = 8.9 Hz, 1H), 4.92 (d, *J* = 8.9 Hz, 1H), 4.10 (d, *J* = 4.1 Hz, 2H), 2.30 (s, 3H), 1.72 (s, 3H), 1.65 (s, 3H). ^13^C-NMR (100 MHz, CDCl_3_) *δ*: 178.1, 176.7, 162.9, 145.7, 139.2, 135.5, 132.6, 130.9, 130.8, 129.4, 129.4, 128.8, 126.9, 125.5, 125.2, 110.6, 83.7, 59.1, 54.8, 27.7, 21.0, 16.0.

*3-Bromo-2,2-dimethyl-4-(4-((p-tolylselanyl)methyl)-1H-1,2,3-triazol-1-yl)-3,4-dihydro-2H-benzo[h]-chromene-5,6-dione* (**21**): Prop-2-yn-1-yl(p-tolyl)selane (230.1 mg, 1.1 mmol) was used. The product was obtained in 26% yield as a yellow powder. m.p. (°C) = 114.1–116.6, IR (solid, cm^−1^) *ν*: 1656 (s), HRMS (EI^+^): 572.0062 [M]^+^. Cald. for [C_16_H_10_SeO_2_]: 572.0083; ^1^H-NMR (400 MHz, CDCl_3_) *δ*: 8.08 (d, *J* = 7.5 Hz, 1H), 7.91 (d, *J* = 7.7 Hz, 1H), 7.73 (t, *J* = 7.5 Hz, 1H), 7.69 (s, 1H), 7.62 (t, *J* = 7.5 Hz, 1H), 7.35–7.24 (m, 4H), 7.18 (t, *J* = 7.0 Hz, 1H), 5.64 (d, *J* = 8.9 Hz, 1H), 4.96 (d, *J* = 8.9 Hz, 1H), 3.81 (s, 2H), 3.75 (s, 2H), 1.75 (s, 3H), 1.67 (s, 3H). ^13^C-NMR (100 MHz, CDCl_3_) *δ*: 178.0, 176.7, 162.8, 144.9, 137.9, 135.4, 134.8, 132.5, 130.9, 130.8, 130.1, 129.4, 125.8, 125.5, 125.1, 110.5, 83.6, 59.1, 54.6, 27.6, 21.3, 21.2, 21.0.

*3-Bromo-2,2-dimethyl-4-(4-((thiophen-2-ylselanyl)methyl)-1H-1,2,3-triazol-1-yl)-3,4-dihydro-2H-benzo[h]chromene-5,6-dione* (**22**): 2-(Prop-2-yn-1-ylselanyl)thiophene (221.3 mg, 1.1 mmol) was used. The product was obtained in 47% yield as a yellow powder. m.p. (°C) = 104.3–108.6, IR (solid, cm^−1^) *ν*: 1656 (s), 710 (w), HRMS (EI^+^): 563.9529 [M]^+^. Cald. for [C_16_H_10_SeO_2_]: 563.9490; ^1^H-NMR (400 MHz, CDCl_3_) *δ*: 8.10 (d, *J* = 7.6 Hz, 1H), 7.90 (d, *J* = 7.6 Hz, 1H), 7.74 (t, *J* = 7.7 Hz, 1H), 7.63 (t, *J* = 7.2 Hz, 1H), 7.43 (s, 1H), 7.37 (d, *J* = 5.3 Hz, 1H), 7.11 (d, *J* = 4.2 Hz, 1H), 6.95 (dd, *J* = 5.2, 3.5 Hz, 1H), 5.58 (d, *J* = 9.0 Hz, 1H), 4.94 (d, *J* = 9.0 Hz, 1H), 4.05 (s, 2H), 1.75 (s, 3H), 1.65 (s, 3H). ^13^C-NMR (100 MHz, CDCl_3_) *δ*: 178.0, 176.6, 162.9, 144.2, 137.2, 135.4, 132.5, 131.7, 130.8, 130.7, 129.4, 128.4, 125.5, 125.2, 123.0, 110.6, 83.6, 59.2, 54.6, 27.7, 24.0, 20.9.

*3-Bromo-4-(4-((butylselanyl)methyl)-1H-1,2,3-triazol-1-yl)-2,2-dimethyl-3,4-dihydro-2H-benzo[h]chromene-5,6-dione* (**23**): Butyl(prop-2-yn-1-yl)selane (192.6 mg, 1.1 mmol) was used. The product was obtained in 31% yield as a yellow powder. m.p. (°C) = 123.1–124.4, IR (solid, cm^−1^) *ν*: 2914 (s), 1670 (s), 782 (w), HRMS (EI^+^): 538.0343 [M]^+^. Cald. for [C_16_H_10_SeO_2_]: 538.0239; ^1^H-NMR (400 MHz, CDCl_3_) *δ*: 8.07 (d, *J* = 7.6 Hz, 1H), 7.91 (d, *J* = 7.7 Hz, 1H), 7.79 (s, 1H), 7.73 (t, *J* = 7.6 Hz, 1H), 7.62 (t, *J* = 7.5 Hz, 1H), 5.65 (d, *J* = 9.0 Hz, 1H), 4.96 (d, *J* = 9.0 Hz, 1H), 3.83 (d, *J* = 3.1 Hz, 2H), 2.57 (d, *J* = 7.3 Hz, 2H), 1.76 (s, 3H), 1.67 (s, 3H), 1.61 (dd, *J* = 15.0, 7.5 Hz, 2H), 1.37 (dd, *J* = 14.4, 7.3 Hz, 2H), 0.89 (t, *J* = 7.3 Hz, 3H). ^13^C-NMR (100 MHz, CDCl_3_) *δ*: 178.0, 162.8, 145.7, 135.4, 130.9, 130.7, 129.3, 125.5, 125.1, 110.6, 83.6, 59.1, 54.8, 32.4, 27.6, 24.2, 23.1, 20.9, 15.4, 13.8.

*3-Bromo-2,2-dimethyl-4-(4-((phenylthio)methyl)-1H-1,2,3-triazol-1-yl)-3,4-dihydro-2H-benzo[h]chromene-5,6-dione* (**24**): Phenyl(prop-2-yn-1-yl)sulfane (163.0 mg, 1.1 mmol) was used. The product was obtained in 47% yield as a yellow powder. m.p. (°C) = 119.5–121.4, IR (solid, cm^−1^) *ν*: 1656 (s), 754 (w), HRMS (EI^+^): 510.0859 [M]^+^. Cald. for [C_16_H_10_SeO_2_]: 510.0482; ^1^H-NMR (400 MHz, CDCl_3_) *δ*: 8.09 (d, *J* = 8.2 Hz, 1H), 7.89 (d, *J* = 7.6 Hz, 1H), 7.73 (t, *J* = 8.2 Hz, 1H), 7.65–7.59 (m, 2H), 7.34 (d, *J* = 7.4 Hz, 2H), 7.29–7.24 (m, 2H), 7.18 (t, *J* = 7.3 Hz, 1H), 5.58 (d, *J* = 9.0 Hz, 1H), 4.89 (d, *J* = 9.0 Hz, 1H), 4.21 (d, *J* = 7.2 Hz, 2H), 1.71 (s, 3H), 1.63 (s, 3H). ^13^C-NMR (100 MHz, CDCl_3_) *δ*: 178.0, 176.6, 162.9, 144.1, 135.4, 135.3, 132.5, 130.9, 130.8, 130.7, 129.4, 129.2, 128.4, 127.0, 125.5, 125.5, 110.5, 83.6, 59.2, 54.6, 29.5, 27.6, 20.9.

#### 3.1.3. Activity Against Cancer Cell Lines

Compounds were evaluated for antitumor activity in cell culture in vitro using several human cancer cell lines obtained from the National Cancer Institute, NCI (Bethesda, MD, USA). The L929 cells (mouse fibroblast L cells NCTC clone 929) employed in this study as a control cell line, were obtained from the American Type Culture Collection (Manassas, VA, USA). All cancer cell lines were maintained in RPMI 1640 medium. The L929 cells were cultivated under standard conditions in DMEM with Earle’s salts. All culture media were supplemented with 10% (cancer and L929 cells) fetal bovine serum, 2 mM l-glutamine, 100 IU/mL penicillin, 100 μg/mL streptomycin at 37 °C with 5% CO_2_. In cytotoxicity experiments, cells were plated in 96-well plates (0.1 × 10^6^ (SF295); 0.7 × 10^5^ (HCT-116); 0.5 × 10^5^ (NCIH-460), 0.1 × 10^6^ (PC3 and L929). All compounds tested were dissolved in DMSO. The final concentration of DMSO in the culture medium was kept constant (0.1%, *v*/*v*). Doxorubicin (0.001–1.10 μM) was used as the positive control, and negative control groups received the same amount of vehicle (DMSO). The cell viability was determined by reduction of the yellow dye 3-(4,5-dimethyl-2-thiazol)-2,5-diphenyl-2*H*-tetrazolium bromide (MTT) to a purple formazan product as described by Mosmann [58]. At the end of the incubation time (72 h), the plates were centrifuged and the medium was replaced by fresh medium (200 μL) containing 0.5 mg/mL MTT. After 3 h, the MTT formazan product was dissolved in DMSO (150 μL) and the absorbance was measured using a multiplate reader (Spectra Count, Packard, ON, Canada). The impact of compounds on cell proliferation was quantified as the percentage of control absorbance of the reduced dye at 550 nm. All cell culture based studies were performed in triplicate. All cells were mycoplasma-free.

## 4. Conclusions

We have demonstrated in previous reports that the strategy of conjoining two redox centers, namely a quinoidal moiety and the atom of selenium or sulfur, represents a promising avenue to prepare new compounds with antitumor activity. Here, we have prepared and assayed 24 such compounds against five cancer cell lines and have identified several such quinone-selenium hybrid molecules with moderate to high activity. Notably, in the past quinone-based triazole chalcogenium-containing agents were considered as potent antitumor derivatives, with IC_50_ values below 1 μM, and we have now added a new class of naphthoquinonoid compounds with improved cytotoxicity against cancer cells. These compounds provide a lead for the further development of redox active and often catalytic molecules based on natural products which eventually may be able to distinguish between cancer and normal cells by modulating the specific pre-existing differences in redox state which for a number of reasons is often found between those cells.

## Figures and Tables

**Table 1:** Cytotoxic activity expressed by IC_50_ μM (95% CI) of compounds in cancer and normal cell lines after 72 h exposure, obtained by nonlinear regression for all cell lines from three independent experiments.

Compounds	HL-60	HCT-116	SF295	NCIH-460	PC3	L929
**2**	1.96 (1.66–2.33)	>10	>10	>10	>10	>10
**3**	4.68 (3.93–5.58)	>10	>10	>10	>10	>10
**4**	2.25 (1.88–2.71)	>10	>10	>10	>10	>10
**5**	2.16 (1.81–2.59)	>10	>10	>10	>10	>10
**8**	>10	>10	>10	>10	>10	>10
**9**	5.39 (3.78–7.73)	>10	>10	>10	>10	>10
**10**	>10	>10	>10	>10	>10	>10
**11**	2.32 (1.72–3.25)	>10	>10	>10	>10	>10
**12**	>10	>10	>10	>10	>10	>10
**13**	>10	>10	>10	>10	>10	>10
**14**	>10	>10	>10	>10	>10	>10
**15**	>10	>10	>10	>10	>10	>10
**17**	0.81 (0.73–0.86)	>8.0	2.60 (2.47–2.72)	2.06 (1.95–2.22)	2.03 (1.90–2.25)	0.52 (0.44–0.81)
**18**	0.59 (0.45–0.70)	0.37 (0.24–0.56)	1.48 (1.34–1.63)	1.32 (1.23–1.53)	1.06 (0.90–1.27)	0.36 (0.30–0.49)
**19**	1.28 (1.16–1.31)	>8.0	1.75 (1.57–1.82)	2.33 (2.23–2.45)	1.55 (1.40–1.94)	0.68 (0.53–0.82)
**20**	1.00 (0.93–1.07)	2.03 (1.79–2.28)	3.12 (2.91–3.24)	3.26 (3.15–3.42)	2.70 (3.15–3.42)	0.61 (0.58–0.68)
**21**	1.00 (0.91–1.03)	>8.0	1.63 (1.42–1.84)	1.49 (1.38–1.59)	1.77 (1.66–1.98)	1.28 (1.19–1.38)
**22**	0.53 (0.48–0.59)	>8.0	2.13 (2.01–2.43)	2.75 (2.54–3.00)	2.47 (2.26–2.68)	3.16 (3.04–3.27)
**23**	0.71 (0.61–0.76)	0.97 (0.88–1.10)	3.43 (3.20–3.69)	2.64 (2.44–2.90)	1.64 (1.55–1.69)	2.12 (1.92–2.27)
**24**	1.94 (1.85–2.08)	4.38 (4.24–4.54)	2.34 (2.16–2.44)	4.91 (4.70–5.26)	2.10 (1.85–2.28)	0.84 (0.71–1.06)
**DOXO**	0.02 (0.01–0.02)	0.21 (0.16–0.29)	0.41 (0.21–0.47)	0.15 (0.13–0.18)	0.76 (0.59–0.93)	1.72 (1.58–1.87)

## Abbreviations

The following abbreviations are used in this manuscript:

ROS	Reactive Oxygen Species
IC_50_	Half Maximal Inhibitory Concentration
CuAAC	Copper(I) Catalyzed Azide-Alkyne Cycloaddition
NBS	*N*-bromosuccinimide
DMF	Dimethylformamide
CI	Confidence Interval
µM	Micromole
NMR	Nuclear Magnetic Resonance
EtOAc	Ethyl Acetate
DMSO	Dimethyl sulfoxide
